# Sidestream Smoke Exposure Increases the Susceptibility of Airway Epithelia to Adenoviral Infection

**DOI:** 10.1371/journal.pone.0049930

**Published:** 2012-11-15

**Authors:** Priyanka Sharma, Abimbola O. Kolawole, Susan B. Core, Adriana E. Kajon, Katherine J. D. A. Excoffon

**Affiliations:** 1 Department of Biological Sciences, Wright State University, Dayton, Ohio, United States of America; 2 Infectious Disease Program, Lovelace Respiratory Research Institute, Albuquerque, New Mexico, United States of America; French National Centre for Scientific Research, France

## Abstract

**Background:**

Although significant epidemiological evidence indicates that cigarette smoke exposure increases the incidence and severity of viral infection, the molecular mechanisms behind the increased susceptibility of the respiratory tract to viral pathogens are unclear. Adenoviruses are non-enveloped DNA viruses and important causative agents of acute respiratory disease. The Coxsackievirus and adenovirus receptor (CAR) is the primary receptor for many adenoviruses. We hypothesized that cigarette smoke exposure increases epithelial susceptibility to adenovirus infection by increasing the abundance of apical CAR.

**Methodology and Findings:**

Cultured human airway epithelial cells (CaLu-3) were used as a model to investigate the effect of sidestream cigarette smoke (SSS), mainstream cigarette smoke (MSS), or control air exposure on the susceptibility of polarized respiratory epithelia to adenoviral infection. Using a Cultex air-liquid interface exposure system, we have discovered novel differences in epithelial susceptibility between SSS and MSS exposures. SSS exposure upregulates an eight-exon isoform of CAR and increases adenoviral entry from the apical surface whilst MSS exposure is similar to control air exposure. Additionally, the level of cellular glycogen synthase kinase 3β (GSK3β) is downregulated by SSS exposure and treatment with a specific GSK3β inhibitor recapitulates the effects of SSS exposure on CAR expression and viral infection.

**Conclusions:**

This is the first time that SSS exposure has been shown to directly enhance the susceptibility of a polarized epithelium to infection by a common respiratory viral pathogen. This work provides a novel understanding of the impact of SSS on the burden of respiratory viral infections and may lead to new strategies to alter viral infections. Moreover, since GSK3β inhibitors are under intense clinical investigation as therapeutics for a diverse range of diseases, studies such as these might provide insight to extend the use of clinically relevant therapeutics and increase the understanding of potential side effects.

## Introduction

Numerous studies have shown that the exposure to secondhand smoke (also called environmental tobacco smoke (ETS), involuntary smoke, and passive smoke) is associated with an increased incidence of lower respiratory tract illness largely resulting from viral infections [1]–[4]. Mainstream smoke (MSS) is the smoke that a smoker inhales from the unlit end of a lit cigarette, while side-stream smoke (SSS) is the smoke that emanates from the tip of the smouldering cigarette. Although ETS is a combination of SSS and exhaled MSS, it is estimated that 50–85% of ETS consists of SSS. Therefore, SSS makes up a significant portion of the smoke that non-smokers encounter [4]–[5]. It is estimated that children exposed to ETS have increased respiratory tract infections resulting in up to 15,000 hospitalizations each year. In adults, acute respiratory infection and chronic obstructive pulmonary disease (COPD) are two of the leading worldwide causes of death [6]. Cigarette smoking is a well-recognized risk factor for viral respiratory infection and is considered a primary cause of COPD [1], [7]. Studies also link chronic adenovirus infection with the development or progression of COPD [8]–[12]. Despite these strong associations, the molecular mechanisms behind the increased susceptibility of the smoke-exposed respiratory tract to viral pathogens are unknown.

Adenoviruses (Ad) are non-enveloped DNA viruses and important causative agents of pediatric respiratory disease, frequently requiring hospitalization, and of epidemic outbreaks of acute respiratory disease in closed communities and among young military recruits during basic training [13]–[14]. Viral entry primarily relies on the presence and accessibility of viral receptors. The Coxsackievirus and adenovirus receptor (CAR) is the primary receptor for many adenovirus serotypes [15]. In polarized epithelia, the apical (air) and basolateral membrane surfaces are divided by tight junctions, which are essential for integrity of the epithelium. It is known that CAR is localized at basolateral junctions in polarized epithelia and that basolateral CAR facilitates adenovirus egress [16]–[17]. However, a major unanswered question is how these pathogenic viruses initiate infection when viruses entering the airway lumen would encounter the apical surface and the receptor is segregated on the basolateral side. We have recently reported that an alternative low abundance eight-exon isoform of CAR, CAR^Ex8^, localizes to the apical membrane of well-differentiated polarized primary human airway epithelia, where it supports apical adenoviral infection [18]. This is a paradigm shift from the commonly held belief that there must be a transient or sustained break in the barrier for the virus to gain access to its receptor. Although increased epithelial permeability resulting from cigarette smoke (CS) exposure is a generally well-accepted phenomenon *in vitro*, this is a transient effect, and many other molecular and structural changes are known to occur after CS exposure [19]–[23].

Based on this information we asked whether CS exposure increases epithelial susceptibility to adenovirus infection and whether this correlates with a change in the abundance of apical CAR. This study utilized a unique *in vitro* system designed to assess the effect of sidestream cigarette smoke (SSS) exposure as compared to mainstream cigarette smoke (MSS) or filtered air (FA) on the susceptibility of polarized respiratory epithelia to viral infection. Using a Walton automatic smoke machine, designed to generate SSS or MSS, coupled to a Cultex air-liquid interface exposure system, we have discovered novel differences in epithelial susceptibility between SSS and MSS exposures that are not discernible by cigarette smoke extract (CSE) exposure model systems. Moreover, we have identified a potential regulatory mechanism that may be responsible for the differences observed between SSS and MSS exposure and may lead to the development of novel therapeutic interventions for individuals experiencing environmental CS exposure.

**Figure 1:**
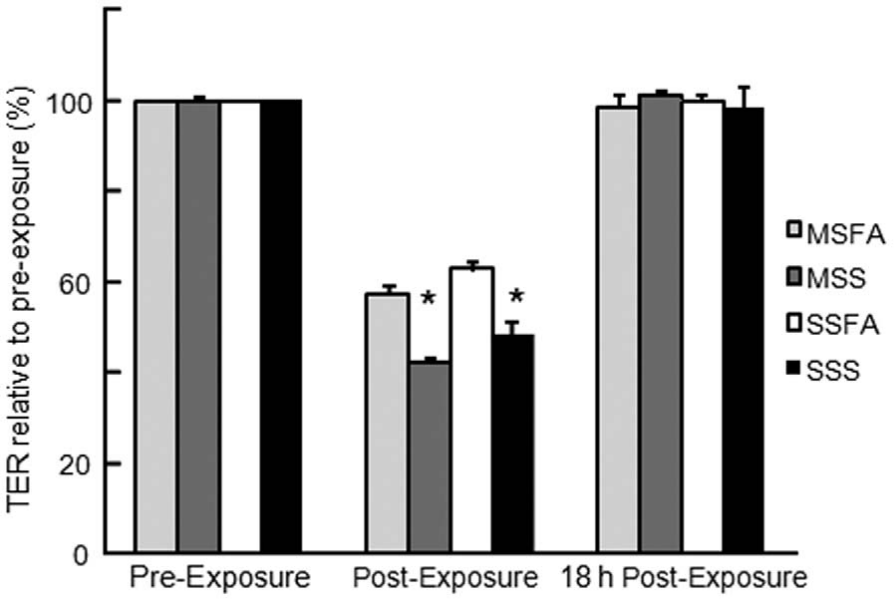
Smoke and air exposure transiently decreases the transepithelial resistance (TER) of polarized CaLu-3 cells. TER recovers by 18 h post-exposure to mainstream cigarette smoke (MSS), sidestream cigarette smoke (SSS) or filtered air (MSFA or SSFA). Data from six replicates per condition and three independent experiments; mean values expressed as a percentage of control+SE of the mean. *p<0.05 MSS or SSS versus pre- or post-exposure and versus MSFA or SSFA respectively.

## Methods

### Cell Culture and Reagents

Human airway epithelial cells (CaLu-3) from ATCC (HTB-55, Rockville, MD) were cultured in growth medium (RPMI 1640 medium (Life Technologies, Grand Island, NY) containing 10 mM HEPES, 10% fetal bovine serum, 1% penicillin/streptomycin and 2 mM L-glutamine). For all smoke exposure experiments, cells were plated in 12 mm Transwell inserts (0.4 µm pore size, Corning Incorporated, Corning, NY) at a density of 5 × 10^5^ cells/well and maintained at 37°C and 5% CO_2_ after plating in growth medium. Medium was changed every 48 h and electrically tight (polarized) epithelial cultures were generally ready by 7 days. The GSK3β inhibitor SB 415286 was obtained from Tocris, (Minneapolis, MN, USA). Cells seeded onto plastic or Transwells were treated overnight with 45 µM of the inhibitor, a dose used in other studies and shown to be specific [24]–[28]. Control cells were treated with vehicle alone (similarly diluted with DMSO).

### Measurement of Transepithelial Electrical Resistance (TER)

Tight junction integrity was assessed by measuring TER using a Millicell ERS meter (Millipore, Bedford, MA), as previously described [17]–[18]. On the seventh day of culture, the medium was changed to exposure medium (RPMI 1640, 10 mM HEPES, 0.1% w/v BSA) and allowed to incubate for one hour at 37°C prior to TER measurement. Cultures exposed to cigarette smoke or filtered air were transferred into maintenance medium (RPMI 1640, 10 mM HEPES, 2% fetal bovine serum, 1% penicillin/streptomycin and 2 mM L-glutamine) and the TER was recorded immediately following exposure and 18 h after exposure.

### Cigarette Smoke Exposure

A Walton automatic smoke machine was used to generate mainstream (MSS) and sidestream (SSS) cigarette smoke, as shown schematically in Figure S1. The operation and characterization of the instrument has been previously reported [21], [29]–[30]. Non-filtered University of Kentucky 2R1 research cigarettes available at the Lovelace Respiratory Research Institute were used to generate smoke for our experimental exposures. Both kinds of smoke (MSS and SSS) were produced by lit cigarettes. In our experimental system, MSS simulated the smoke that is inhaled by a smoker from the unlit end of a burning cigarette and was collected in the MSS collector (Figure S1). SSS was collected in the SSS collector so that it simulated the smoke that comes off the smoldering end. Cigarettes were maintained between 30% and 40% relative humidity at least two days prior to use. One 2R1 cigarette was smoked per exposure for 8 minutes by repeating cycles of smoke for 2 s, hold for 28 s, and purge for 30 s for a total of 8 puffs for MSS. Three 2R1 cigarettes were smoked in the same exposure cycle to generate SSS. MSS and SSS exposure were normalized to total nicotine content delivered as previously described [21]. The exposure cycles closely approximate a human smoker alternating between puffs from the cigarette and breaths of filtered air. Air used for smoking or for purging of the smoke reservoir was humidified to 100% relative humidity before use by passing it through a bubbler. The Cultex exposure apparatus (Vitrocell, Hannover, Germany) [21] was hooked directly to the Walton Smoke machine, as shown in Figure S1, and provides a unique means of performing air-liquid interface exposures which more closely resemble *in vivo* conditions than other “wet” exposure systems, such as smoke condensates [31]–[32]. Polarized epithelial cells seeded on Transwells with transepithelial electrical resistance (TER) between 1900 Ωcm^−2^ and 2000 Ωcm^−2^ were allowed to equilibrate for 60 min in exposure media. Transwell cultures were placed in the Cultex system maintained at 37°C and exposed to SSS or MSS or filtered room air as control (FA). Control FA exposures were performed at the same time as SSS or MSS exposures and are termed SSFA or MSFA to indicate coupled experimental controls. Immediately before exposure, TER was measured, and then all but 50 µl of exposure medium was removed from the apical compartment. Cultures were placed into water-jacketed temperature-controlled glass Cultex chambers (three inserts in each chamber), with exposure medium in contact with the basolateral side of the culture. Ports above the apical surface delivered and removed exposure mixtures to the experimental wells or FA to control wells from the apical surfaces of the Transwell cultures. Exposure flow was independently controlled for each exposure chamber by individual MassTrak in-line flow controllers (Automatic Controls, Wixom, MI) connected to a vacuum reservoir on the post-exposure side. Flow rate for all exposures was maintained at 25 ml/min/chamber (8.3 ml/min/Transwell). Following exposure, the Transwell cultures were returned to plates containing maintenance medium.

**Figure 2:**
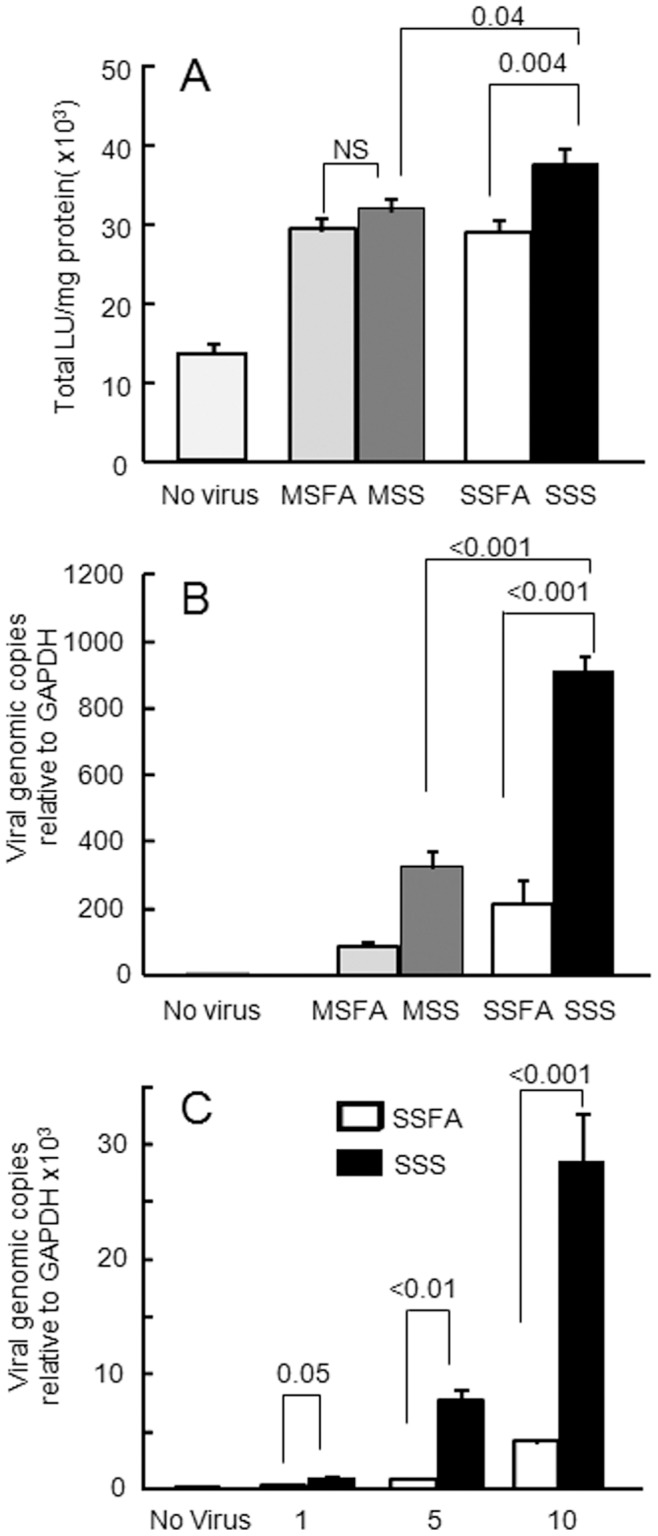
Sidestream cigarette smoke (SSS) exposure increases epithelial susceptibility to adenovirus infection (entry and transduction). Polarized CaLu-3 cells were infected from the apical surface with hAdV5-β-gal (MOI 10 pfu/cell) or mock infected (MOI 0) 18 h post-exposure to air (MSFA or SSFA), mainstream cigarette smoke (MSS), or SSS. Cells were evaluated for A: β-Galactosidase activity (transduction, four to six replicates per condition; three independent experiments) or B: viral genomes (viral entry) 24 h post-infection (four biological replicates per condition measured in duplicate in each qPCR assay; three independent experiments). C: Intracelluar viral genome load 24 h after apical adenovirus infection, at increasing MOI, in SSS- or SSFA-exposed epithelia. Results for MSS, SSS, and FA without virus were identical and were combined for graphical representation (No Virus; four replicates per condition; two independent experiments). Representative experimental results are shown as mean+SE.

**Figure 3:**
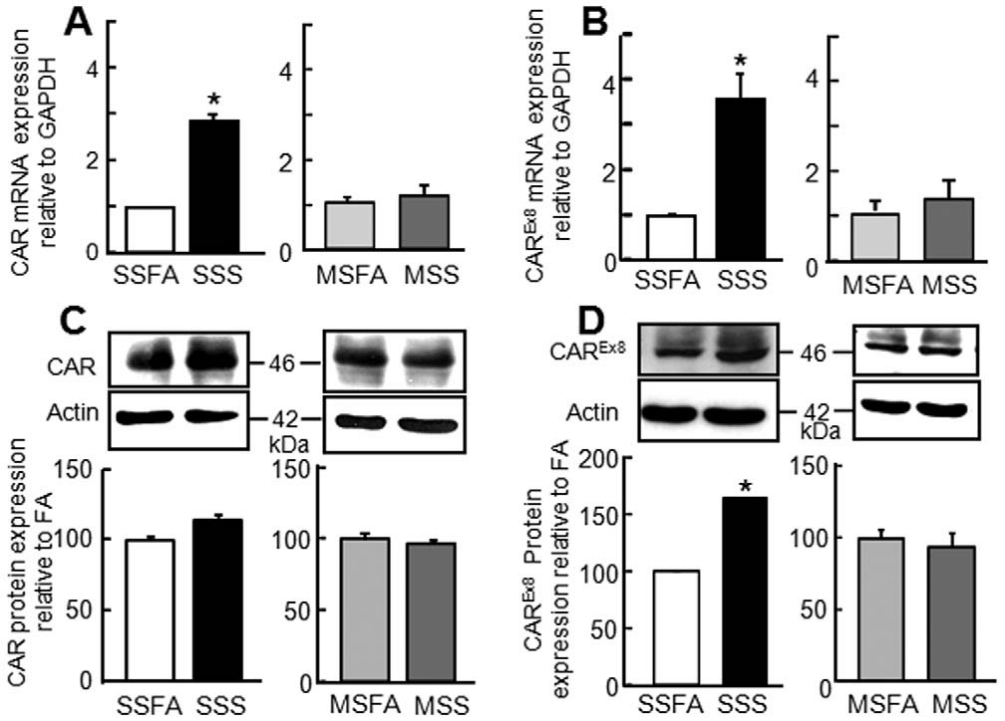
CAR expression is increased in polarized CaLu-3 cells 18 h post-sidestream cigarette smoke (SSS) exposure relative to mainstream cigarette smoke (MSS), or filtered air (SSFA or MSFA) exposure. A: Total CAR mRNA (three to four biological replicates per condition measured in duplicate in each qPCR assay; three independent experiments) and B: CAR^Ex8^ mRNA (three to four biological replicates per condition measured in duplicate in each qPCR assay; three independent experiments) quantification using quantitative RT-PCR. Mean values of three independent experiments relative to control+SE of the mean. C: Total CAR and D: CAR^Ex8^ protein and corresponding β-actin expression by Western blot (representative) and Multi-Guage image analysis (mean values from three independent experiments expressed as a percentage of control+SE of the mean). *p<0.05.

### Viral Infection

Polarized CaLu-3 cells, exposed to either smoke or filtered air, were incubated in maintenance medium for 18 h prior to apical infection with a HAdV-5 vector containing the β-galactosidase gene (Ad-β-Gal, University of Iowa Vector Core, Iowa City, IA), diluted to 100 µl with PBS, at different multiplicities of infection (MOI) as described in the results section, for 1 h at 37°C. The inoculum was then removed, cells were rinsed with phosphate buffered saline (PBS), and maintenance medium was replenished. Cells were lysed 24 h later and β-galactosidase expression and protein concentration were determined as previously described (Galacto-Light Plus System, Applied Biosystems, USA; Bio-Rad Protein Assay, Bio-Rad, CA, USA) [18].

### Western Blotting

Smoke and air exposed CaLu-3 cells were harvested 18 h post-exposure. Samples were washed with ice-cold PBS, and lysed in buffer (50 mM Tris pH 7.4, 137 mM NaCl, 1% Triton X-100, 5 mM EDTA, 1 mM EGTA, 1 mM NaF, 1 mM Na_2_VO_4_, protease inhibitors (10 µg/ml) leupeptin, aprotinin, pepstatin, and 1 mM phenylmethylsulfonyl fluoride) by rocking at 4°C. Cells were scraped, sonicated five times with five pulses and centrifuged at 14,000 g for 10 minutes in a microcentrifuge. The supernatant was transferred to fresh tubes and protein concentration was determined with the Bio-Rad protein assay (Bio-Rad). Equal amounts of protein were subjected to 10% polyacrylamide gel electrophoresis. Gels were transferred to a polyvinylidene difluoride (PVDF) membrane (Millipore, Bedford, MA), blocked with 5% BSA, washed, probed with primary antibodies for CAR (1605p (total CAR), 5678 (CAR^Ex8^) as described previously [18], [33], GSK3β and GSK3βpS9 (Cell Signaling, Danvers, MA), or β-actin (Millipore, Billerica, MA), washed and incubated with HRP conjugated secondary antibodies (Jackson Immuno Research, West Grove, PA). Band detection with ECL reagents (Pierce, Rockford, IL) was imaged on a Fuji LAS 4000 and the intensity of the bands was measured with Multi Gauge software (Fujifilm, Tokyo, Japan). All densitometry data was normalized to β-actin protein levels as a loading control and the percent change was calculated relative to control samples. All graphs represent calculated averages from a minimum of three individual experiments.

### RNA Isolation, Reverse Transcription, Real-time PCR and Adenoviral Genome Quantification

To investigate changes in gene expression, total RNA was isolated from cigarette smoke- or air-exposed CaLu-3 cells and CaLu-3 cells treated with SB415286 using TRIzol (Life Technologies, Grand Island, NY) according to manufacturer’s protocol. cDNA was synthesized from 1 µg of RNA using Quanta First Strand Kit (Quanta BioSciences,Gaithersburg, MD) prior to quantitative PCR (qPCR) according to the manufacturer’s instruction. For hAdV5 genome quantification, total DNA was purified from the lysates of adenovirus-infected cells using the DNeasy Blood and Tissue kit (QIAGEN, Valencia, CA) according to the manufacturer’s instructions. DNA was eluted with 100 µl of Qiagen AE elution buffer. qPCR was performed using SYBRG with low ROX (Quanta, Gaithersburg, MD) in Stratagene’s Real Time PCR System (Agilent Technologies, Santa Clara, CA) using primers for glyceraldehyde 3-phosphate dehydrogenase (GAPDH) or β-actin mRNA as internal standards. The relative expressions of target genes were quantified using comparative C_t_ analysis by using Mx4000p software v5 for data analysis. Primers used were: CAR-F: TCGGCAGTAATCATTCATCCCTGG, CAR^Ex8^-R: ACTGTAATTCCATCAGTCTTGTAAGGG [18], totalCARF: TACAGTCAGAAACAGAGTGGGC, total CAR-R: CCAGCTTTATTTGAAGGAGGGAC GSK3β-F: GGTCTATCTTAATCTGGTGCTGG, GSK3β-R: TGGATATAGGCTAAACTTCGGAAC adenovirus hexon gene specific primers AdqPCR-F: ACGCCTCGGAGTACCTGAG and AdqPCR-R: GTGGGGTTTCTGAACTTGT [34]. Abundance relative to GAPDH gene expression was calculated for each gene of interest. GAPDH-F: CACCCTGTTGCTGTAGCCAAA, GAPDH-R: CAACAGCGACACCCACTCCT. qPCR efficiency was comparable for all primer pairs and ranged from −3.0 to −3.4.

**Figure 4:**
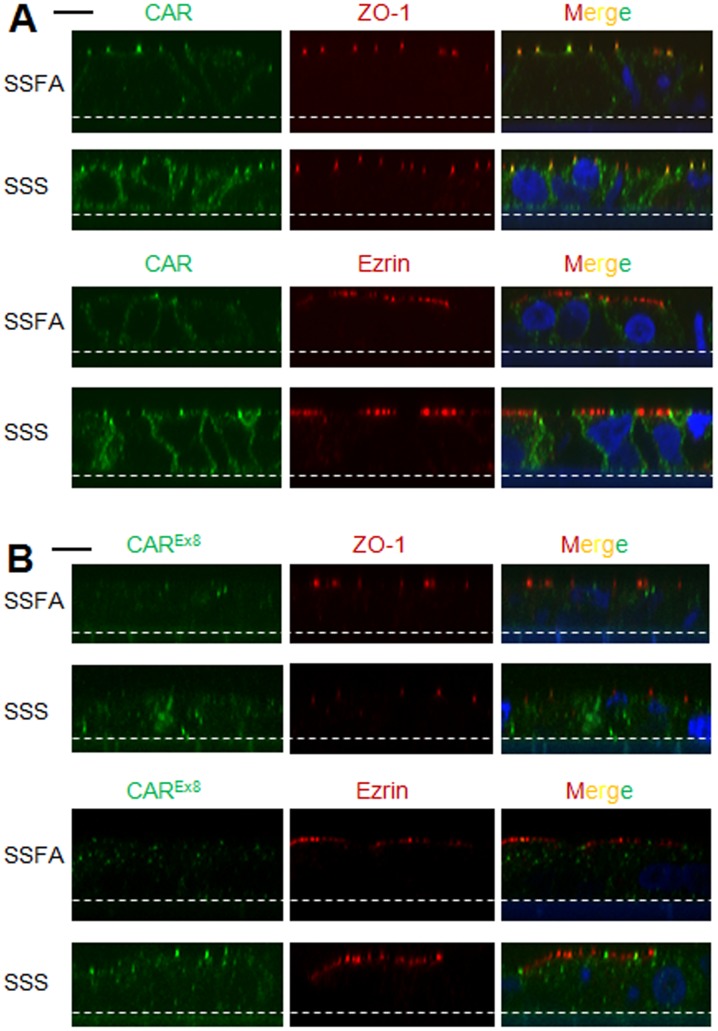
CAR expression is increased and localization is altered in polarized CaLu-3 cells 18 h post-SSS exposure. Immunofluorescence staining of A) total endogenous CAR (green) and B) CAR^Ex8^ (green), co-stained with antibodies directed against either the tight junction protein ZO-1 (red) or the apical protein ezrin (red), in polarized CaLu-3 cells 18 h after exposure to SSFA or SSS. Nuclei are counterstained with DAPI (blue). X–Z sections representative of three independent experiments are shown. Dotted white line represents the Transwell filter that cells are seeded on. Black line = 10 µm. Confocal microscopy (60× oil immersion).

**Figure 5:**
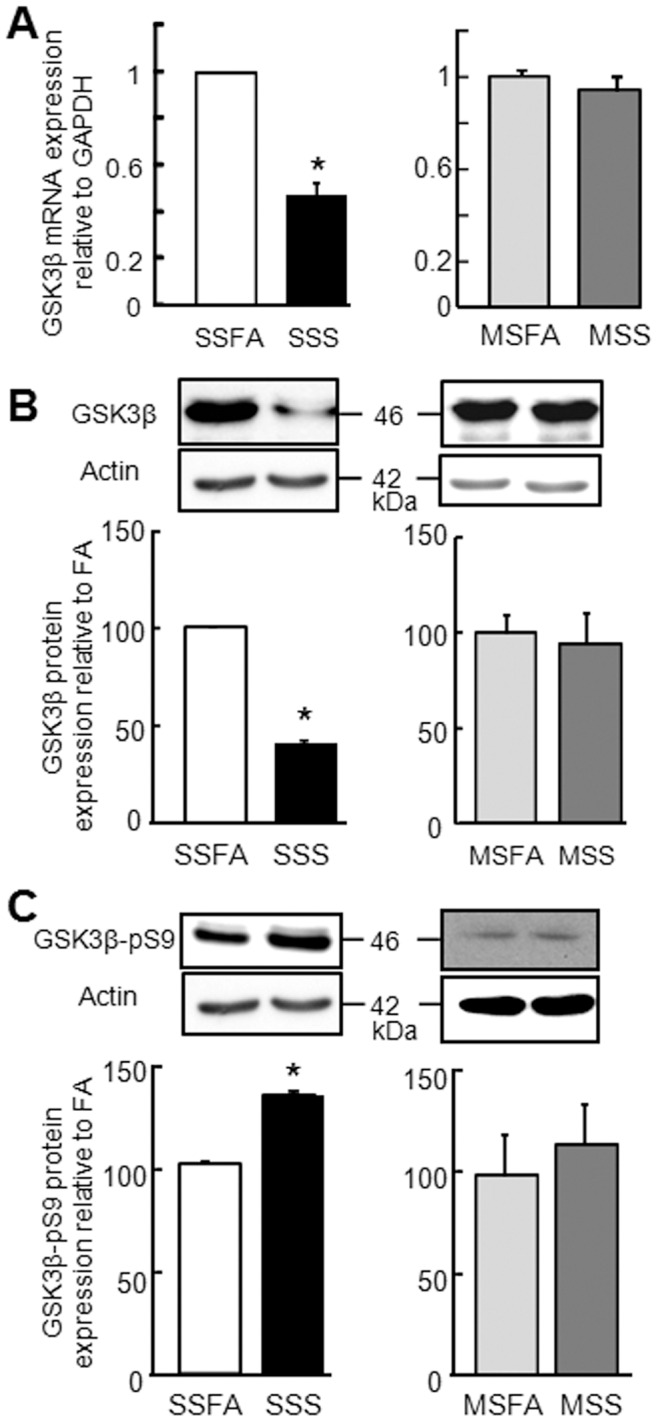
GSK3β is downregulated 18 h post- sidestream cigarette smoke (SSS) exposure in comparison to air (SSFA or MSFA) or mainstream cigarette smoke (MSS) exposure. A: Analysis of total GSK3β mRNA levels by quantitative RT-PCR (four to six biological replicates per condition measured in duplicate in each qPCR assay; three independent experiments; mean values from three independent experiments relative to control+SE of the mean). B: GSK3β and C: GSK3β-pS9 protein levels, representative Western blot and densitometric analysis, relative to β-actin (mean values from three independent experiments (duplicate gels per experiment) expressed as a percentage of control+SE of the mean). *p<0.05.

### Immunocytochemistry

CaLu-3 cells seeded on Transwell inserts, as above, were washed once with PBS, fixed with 4% paraformaldehyde, permeabilized with 0.1% Triton X-100, and blocked with 2% BSA in SuperBlock (Pierce, Rockford, IL), as previously described [17]–[18]. Epithelial cultures were incubated with primary CAR specific antibodies (1605 p (total CAR), 5678 (CAR^Ex8^)) and antibodies for the tight junction protein zona-occludens (ZO-1) or apical surface-associated protein ezrin (Santa Cruz Biotechnology, Santa Cruz, CA), washed extensively with PBS, and incubated with goat anti-rabbit Alexa-488 or anti-mouse Alexa-568 secondary Ab. After washing, slides were coverslipped with Vectashield mounting media (Vector Laboratories, Inc, Burlingame, CA). Staining was evaluated by laser scanning confocal microscopy (Olympus FV 1000) at 60 × magnification (oil immersion); images are shown as either single X–Y or X–Z sections.

### Cell Surface Biotinylation

CaLu-3 cells were seeded at 1×10^6^ cells per 10 cm dish and allowed to reach confluency. Cells were treated with SB415286 or control vehicle for 18 h prior to incubation with Sulfo-NHSSS-biotin 1 mg/ml (Thermo Scientific, Rockford, IL) for 1 h at 4°C with rocking, as previously described [33]. Briefly, after washing, free Sulfo-NHS-SS-biotin was quenched with 100 mM glycine for 20 min at 4°C. The cells were washed three times with PBS (including Ca^2+^and Mg^2+^) and lysed with lysis buffer (50 mM Tris pH 7.4, 150 mM NaCl, 1% Triton X-100, protease inhibitors (10 µg/ml) leupeptin, aprotinin, pepstatin, and 1 mM phenylmethylsulfonyl fluoride). Cells were then scraped, lysates collected, and sonicated with five pulses. This was followed by centrifugation at 14,000 *g* at 4°C for 15 min. NeutrAvidin beads (Thermo Scientific, Rockford, IL) were added to the supernatant and incubated at 4°C for more than 2 h with rotation. NeutrAvidin beads were then collected by centrifugation at 1,000 g at 4°C for 3 min and washed three times with ice-cold wash buffer. The sulfo-NHS-SS-biotin-labeled proteins were eluted from NeutrAvidin beads with SDS-PAGE sample buffer at 100°C for 10 min. This was followed by SDS-PAGE and Western blot using CAR specific antibodies (1605 p (total CAR), 5678 (CAR^Ex8^)) and antibodies for the tight junction protein occludin (Life Technologies) or apical surface protein DAF (BD Bioscience, San Jose, CA).

**Figure 6:**
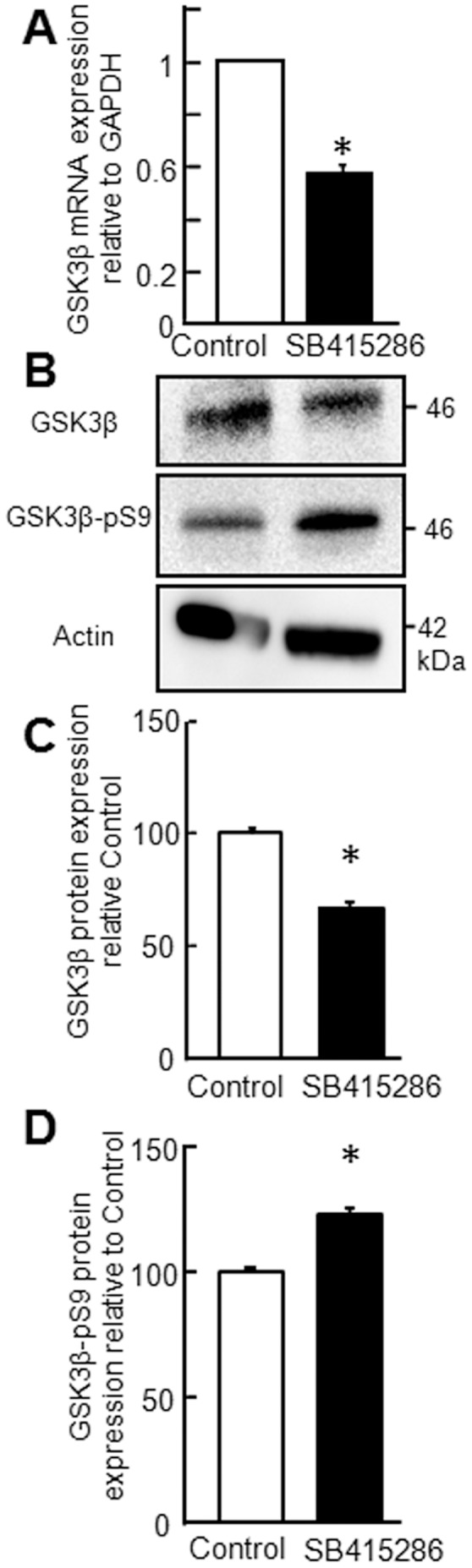
GSK3β is downregulated in polarized CaLu-3 cells 18 h post-GSK3β inhibitor (SB415286) treatment. A) Total mRNA expression of GSK3β in control (white) or SB415286 (black) treated CaLu-3 epithelia (four biological replicates per condition measured in duplicate in each qPCR assay; mean values from three independent experiments relative to control+SE of the mean). B) Representative Western blot analysis of GSK3β, GSK3β-pS9, and β-actin protein levels. Quantification of C) GSK3β or D) GSK3β-pS9 protein levels, relative to β-actin (mean values from three independent experiments expressed as a percentage of control+SE of the mean). *p<0.05.

### Statistical Analysis

All experiments were performed at least three times. Sidestream and mainstream exposures were performed sequentially, ensuring a complete system purge between exposures, on the same day. In each experiment triplicate samples from all exposure types were collected. Microsoft Excel and Graph Pad Prism V5 (La Jolla,CA) were used to perform statistical analyses. Significant differences were analyzed using student’s t test and two-tailed distribution. Results were considered to be statistically significant if p<0.05. The D’Agostino-Pearson omnibus test (Prism) was used to confirm data normality (p>0.05).

**Figure 7:**
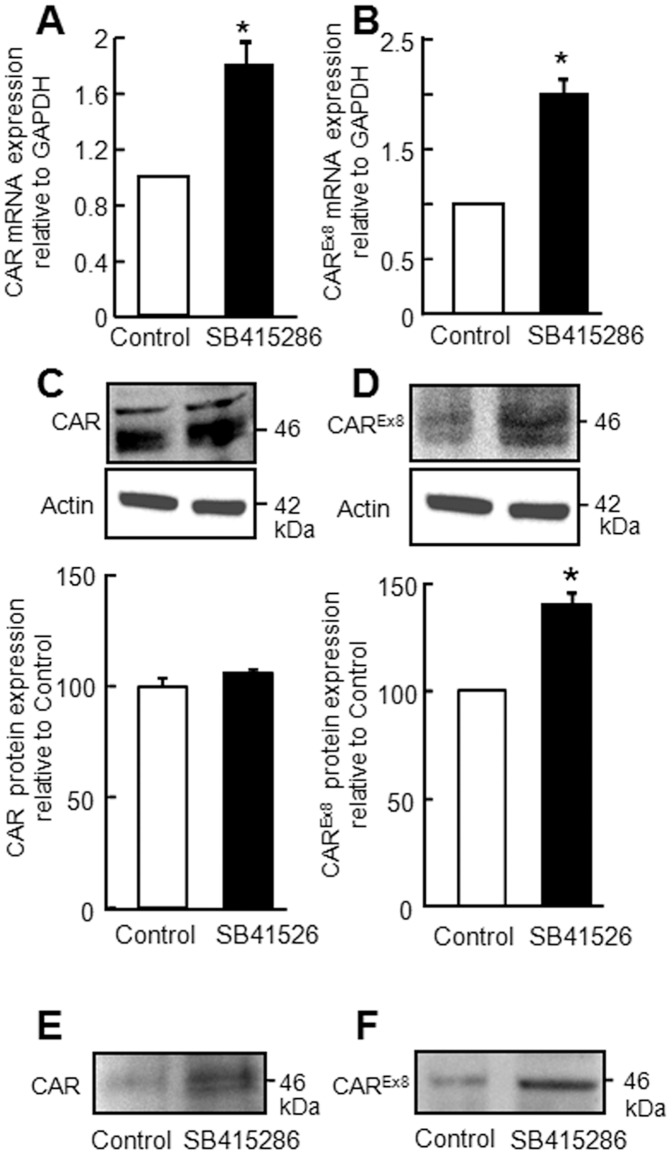
CAR expression is upregulated in polarized CaLu-3 cells 18 h post apical treatment with 45 µM of GSK3β inhibitor (SB415286). A: Total CAR and B: CAR^Ex8^ mRNA levels by quantitative RT-PCR (three biological replicates per condition measured in duplicate in each qPCR assay; mean values from three independent experiments relative to control+SE of the mean) and C: total CAR and D: CAR^Ex8^ and corresponding β-actin protein expression by Western blot (representative) and quantification using Multi-Guage densitometric analysis (mean values from three independent experiments expressed as a percentage of control+SE of the mean). Apical biotinylation of polarized CaLu-3 cells 18 h post-treatment with 45 µM SB415286 shows increased protein levels of E: total CAR and F: CAR^Ex8^ specifically (representative blot shown from three independent experiments). *p<0.05.

## Results

### Cigarette Smoke Exposure and Tight Junction Integrity

In order to determine whether the tight junctions of cultured human airway epithelial cells (CaLu-3) were compromised upon exposure to smoke, CaLu-3 cells were seeded on semi-permeable membranes and allowed to polarize for 7 days, at which time they acquired an average TER of 1916±42 Ω/cm^2^. A significant drop in TER was observed immediately after the apical surface of each culture was exposed to cigarette smoke (CS; MSS or SSS; ∼900 Ω/cm^2^) and filtered air (FA; MSFA or SSFA; ∼1200 Ω/cm^2^) (Figure 1), indicating that some changes occurred as a result of the experimental procedures. No TER drop was observed in non-exposed cells (data not shown). Although the drop in TER was significantly greater, and similar, for both SSS and MSS exposure conditions in comparison to FA, disruption was transient and all cultures completely recovered TER by 18 h post exposure.

### SSS-exposure Enhanced Adenovirus Entry

It is well known that viral infection is increased when tight junction integrity is compromised [16]–[17]. Thus, we asked whether smoke exposure altered the susceptibility of epithelial cells to adenovirus infection after the epithelial junctional integrity was fully recovered, as observed at 18 h. The apical surfaces of SSS, MSS, or FA exposed cultures were inoculated with Ad-β-Gal at a MOI of 10 pfu/cell 18 h post-exposure when the junctional integrity was recovered. Cells were analyzed for two different measures of viral entry, β-galactosidase activity (transduction) and viral genome load (entry), 24 h later. Both viral transduction (Figure 2A) and genome load, relative to GAPDH, (Figure 2B) were significantly increased in SSS-exposed cultures as compared to air or MSS. Although a modest increase in viral genome copy number was observed in MSS-exposed cultures as compared to MSFA, only SSS-exposed cultures exhibited a significant increase both in viral transduction and genome copy number. Furthermore, an increase in transduction for SSS-exposed epithelia, relative to sidestream filtered air (SSFA) exposed epithelia, was noted with increasing MOI (Figure 2C). This increase in virus transduction in SSS exposed cells could be due to several factors, such as increased receptor levels, increased accessibility to viral receptor, increased endocytosis, or a combination of factors. In order to address the mechanisms of increased entry and transduction, we first investigated the levels and localization of the primary receptor, CAR.

**Figure 8:**
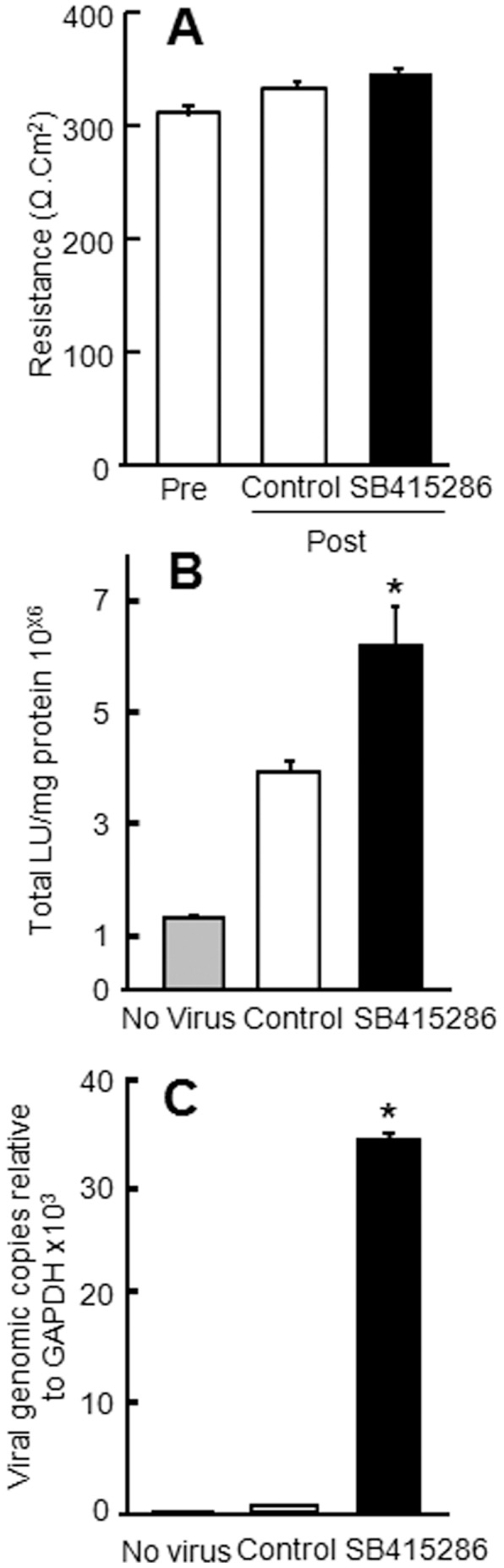
Apical entry and transduction of hAdV5-β-gal is increased in polarized CaLu-3 cells 18 h post apical treatment with 45 µM of GSK3β inhibitor (SB415286). A: TER does not change in polarized CaLu-3 cells treated with GSK3β inhibitor, SB415286, for 18 h. B: A significant increase in adenoviral transduction is observed upon treatment. C: A significant increase in intracellular adenovirus genome copies occurs upon treatment. Representative data shown; mean of four replicates per experiment; three independent experiments. *p<0.05 SB415286 versus control or no virus.

**Figure 9:**
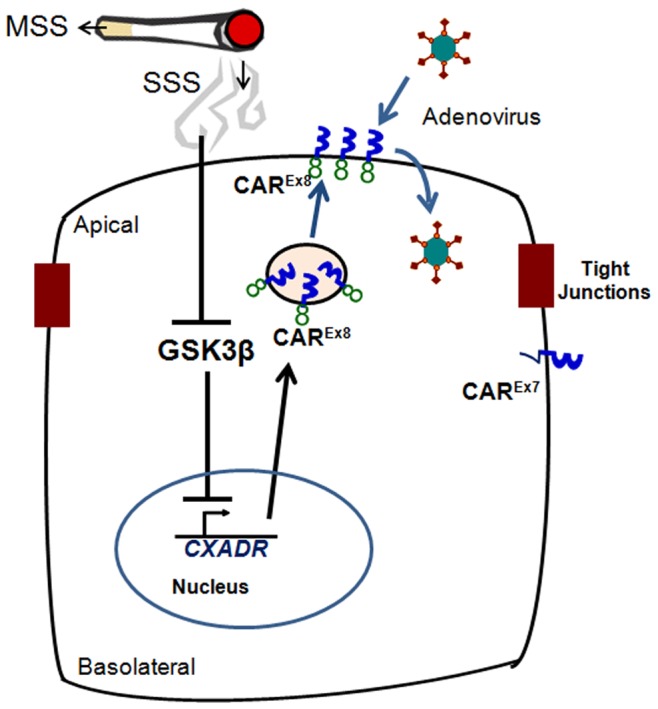
Schematic representation of the proposed mechanism for increased epithelial susceptibility to adenovirus infection upon sidestream cigarette smoke (SSS) exposure. SSS downregulates GSK3β protein expression, which leads to transcriptional upregulation of CAR^Ex8^, leading to increased susceptibility of the epithelium to adenovirus infection from the apical surface. *CXADR*, CAR gene.

### SSS Exposure Results in Increased CAR^Ex8^ Expression

We have recently identified two different isoforms of CAR, CAR^Ex7^ and CAR^Ex8^, that localize to distinct cellular compartments in polarized primary airway epithelia [18]. CAR^Ex7^ is known to be present at basolateral junctions of polarized cells and hence inaccessible for virus binding. CAR^Ex8^ appears to be present at the apical surface of polarized primary human airway epithelia and therefore is potentially available to mediate viral binding and entry. The ratio of CAR^Ex7^ to CAR^Ex8^-specific transcripts in CaLu-3 cells is roughly 12∶1 (data not shown), and this is in agreement with the 10-fold ratio observed in primary airway [18]. Using qRT-PCR primers designed to quantify total CAR or CAR^Ex8^ mRNA specifically, we observed a significant increase in both CAR and CAR^Ex8^ transcript levels in samples extracted 18 h post-exposure in SSS-exposed CaLu-3 cells in comparison to SSFA. In contrast, no change was observed in MSS-exposed samples as compared to MSFA exposure (Figure 3A, B). Interestingly, WB with a CAR-specific Ab that detects total CAR (1605 p; Figure 3C) [17] did not demonstrate an increase in total CAR, relative to actin, in SSS- or MSS-exposed cells (Figure 3C). However, a significant increase in CAR^Ex8^ protein level was detected with a CAR^Ex8^-specific antibody (5678 p, Figure 3D) after SSS but not MSS exposure. Our ability to detect a difference in the levels of CAR^Ex8^ protein but not total CAR may reflect the high level of CAR^Ex7^ protein relative to CAR^Ex8^ at baseline, the length of time required to synthesize CAR, or the sensitivity of our antibody. These data suggest that SSS exposure causes an increase in newly transcribed and synthesized CAR^Ex8^. To investigate whether there was a change in the localization of CAR, we stained SSS or SSFA exposed cells with antibodies directed against total CAR (green; Figure 4A) or CAR^Ex8^ (green; Figure 4B). The epithelia were counterstained with antibodies directed against the tight junction protein ZO-1 (red) or the apical protein ezrin (red). There was a striking difference in the amount and localization of CAR^Ex8^ between SSFA and SSS exposure (Figure 4B). Although much of the CAR^Ex8^ staining after SSS exposure was diffuse within the cell, and may have also been at the basolateral junctions between the cells, more CAR^Ex8^ appeared to co-localize with ezrin at the apical surface than in SSFA control (Figure 4B) or in baseline conditions (data not shown). This is consistent with the diffuse localization of exogenous CAR^Ex8^ in primary airway epithelia [18]. Interestingly, there appeared to be an increase in total CAR (Figure 4A), which may reflect the increase in CAR^Ex8^. In contrast, neither MSS nor MSFA exposures significiantly altered total CAR or CAR^Ex8^ localization (Figure S2, also see representative Figure 2A). Taken together, SSS exposure enhanced adenovirus entry through the upregulation of primary viral receptor, and the CAR^Ex8^ isoform in particular. These data led us to investigate potential mechanisms behind the increased expression of CAR^Ex8^ in SSS-exposed cells.

### SSS Exposure Downregulates GSK3β

Glycogen synthase kinase 3β (GSK3β) is a ubiquitously expressed constitutively active serine/threonine kinase that regulates multiple signaling pathways, thereby controlling a broad spectrum of cellular responses, including metabolism, gene transcription, protein translation, cell-cycle regulation, and apoptosis [35]. In particular, inhibition of GSK3β causes the upregulation of the transcriptional regulators Sp1, SNAIL, and SMAD [25], [27], [36]. Recent evidence suggests that the gene for CAR, *CXADR*, is regulated by Sp1, SNAIL, and SMAD [37]–[39]. Moreover, previous studies have shown GSK3β is downregulated when lung carcinoma cells are exposed to cigarette smoke extract [40]–[41]. To determine whether SSS or MSS exposure resulted in GSK3β downregulation, total RNA or protein lysates were prepared 18 h post-SSS, -MSS or -FA exposure. Quantitative RT-PCR (qRT-PCR) and WB with anti-GSK3β antibodies showed a significant decrease in both the mRNA and protein levels of GSK3β after SSS but not MSS exposure (Figure 5A, B). Additionally, GSK3β is known to be negatively regulated by phosphorylation at amino acid serine 9 (GSK3β-pS9). Thus, we asked whether there was a shift in the levels of inactive GSK3β-pS9. WB of samples after SSS exposure, but not MSS, demonstrated significantly higher levels of GSK3β-pS9 using a phospho-S9 specific antibody (Figure 5C). These data indicate that SSS exposure not only reduces GSK3β transcript and total protein levels but also results in the inhibition of GSK3β.

### GSK3β Inhibition Increases Apical CAR^Ex8^ and Adenovirus Infection

To determine whether we could inhibit GSK3β and recapitulate the same outcome as SSS exposure, a highly specific and potent inhibitor of GSK3β, SB415286 [42], or vehicle (control), was applied to the apical surface of polarized CaLu-3 cells for 18 h. The epithelial cells were then lysed and subjected to qRT-PCR for GSK3β, and WB for GSK3β and GSK3β-pS9 (Figure 6). The levels of GSK3β were significantly lower at both mRNA and protein levels after inhibition (Figure 6A–C), relative to control. Inhibition of GSK3β also increased GSK3β-pS9 (Figure 6B, D). These results were similar in magnitude to the samples exposed to SSS (Figure 5).

To further determine the effect of GSK3β inhibition on total CAR and CAR^Ex8^, the mRNA and proteins levels of CAR (Figure 7A, C) and CAR^Ex8^ (Figure 7B, D) were analyzed by qRT-PCR and WB. Similar to SSS-exposed samples (Figure 3A, C), we observed an increase in total CAR and CAR^Ex8^ at the mRNA level, while only CAR^Ex8^ was significantly increased at the protein level. Since viral infection requires accessible apical receptor, the levels of CAR and CAR^Ex8^ at the apical surface were investigated by apical surface biotinylation. Sulfo-NHSSS-biotin analog, which is membrane impermeable, was used to specifically label and isolate apical membrane-associated proteins. Interestingly, an increase in total CAR and CAR^Ex8^ at the apical surface was observed as compared to the control samples (Figure 7E, F, respectively). We next asked whether epithelial susceptibility to adenovirus infection was increased upon GSK3β inhibition. No difference in TER was observed between SB415286 and vehicle treated epithelia (Figure 8A). Epithelia were infected from the apical surface with Ad-β-Gal at a MOI of 0 or 125 pfu/cell and both transduction (Figure 8B) and intracellular viral genome load (Figure 8C) were significantly increased 24 h post-infection. These results were of a similar magnitude to those obtained for the samples exposed to apical SSS demonstrating that GSK3β inhibition alone is sufficient to increase the susceptibility of polarized CaLu-3 cells to apical adenoviral infection.

## Discussion

There is a significant body of data that supports the association between tobacco smoking and increased signs and symptoms of viral respiratory tract infection and disease in children and adults [1], [7], [13]. Sidestream smoke (SSS) is the smoke that comes off of the tip of the burning cigarette between puffs and constitutes approximately 50–85% of secondhand tobacco smoke [4]–[5]. In this study we have utilized an *in vitro* exposure system to partially recreate ETS exposures and examine their effect on viral infection of polarized lung epithelial cells. We have found that adenoviral infection of SSS-exposed polarized CaLu-3 cells is increased compared to MSS- and air-exposed cells. Surprisingly, no significant difference in the magnitude of apical viral infection was observed between MSS or air exposure. Previous exposure to viral pathogens and the level of pre-existing immunity, as well as smoke-induced immunosuppression, are thought to be key factors contributing to the susceptibility of the airway to viral infection in individuals exposed to CS. Our data indicate that there are additional underlying molecular mechanisms inherent to the response of epithelial cells and that the composition of SSS is unique in activating these mechanisms.

These data provide rationale for investigating the identity of components present in SSS and MSS [43]–[44]. It is possible that one or more of the components found in SSS but not in MSS may be required to induce the cellular changes leading to increased epithelial susceptibility to adenovirus infection. Furthermore, the identification of the activating component(s) of SSS may contribute to a better understanding of the mechanisms of action of other complex environmental pollutants known to alter epithelial biology [31]–[32].

We have found that increased adenoviral infection is associated with a significant increase in the levels of the primary adenovirus receptor, CAR, and in particular CAR^Ex8^, upon acute SSS exposure. Increased viral infection has previously been associated with disruption of the epithelial junctional barrier, as determined by TER [16]–[17]. TER is a sensitive measure of barrier integrity that can reflect membrane channel opening or closing, cellular swelling, shrinking, edge effects, or simply the disruption of a few cellular junctions that dominate the circuit [45]. Several lines of evidence indicate that the CaLu-3 epithelial cultures used in these studies were well polarized 18 h post smoke exposure, including high TER, limited apical viral infection, and distinct immunolocalization of proteins known to be apical (ezrin) or within the tight junction (ZO-1), and the CAR isoforms. We have previously shown that the CAR^Ex8^ isoform does not localize at the basolateral surface, and when apically localized, it is available for viral entry from the apical surface of airway epithelia [18]. Consistent with diffuse localization upon overexpression in primary airway cells, our immunocytochemistry data suggests that newly translated endogenous CAR^Ex8^ may end up distributed throughout the epithelial cells, in addition to the apical surface (Figure 4, 7F). Although both active and passive smokers are exposed to SSS, Shaykhiev *et al.* have recently reported that CAR expression is downregulated in the airways of both healthy smokers and people with COPD, relative to non-smokers [23]. It is possible that in active smokers the levels of CAR are constitutively downregulated due to chronic exposure by a mechanism such as epigenetic modification, and are not susceptible to further regulation by the acute effects of SSS. Immunological factors may also play a greater role in epithelial susceptibility to adenovirus infection in smokers compared to non-smokers. Further investigation is required to validate our observations in primary human airway cells, and to identify the combined effects of SSS and MSS on viral infection, as well as the effects at later time points and upon repeated or chronic exposure. Future research should also address whether the infectivity of other respiratory viruses that utilize apical receptors, such as sialic acid (e.g. influenza and AAV5)[46]–[48], is similarly affected by SSS. Epidemiological evidence clearly shows that children and infants exposed to secondhand smoke are more prone to viral infection as compared to non-exposed children. Therefore, understanding the mechanism behind upregulation of CAR, and CAR^Ex8^ in particular, may facilitate the development of novel therapeutics to limit viral infection in these susceptible individuals.

Our data show that SSS inhibits GSK3β by reducing total GSK3β expression at both the mRNA and protein levels, as well as increasing the levels of inactive phosphorylated-GSK3β. Although there are two known isoforms of GSK3, α and β, it is well documented that GSK3β is the developmentally essential form and that it regulates the stability or activity of many diverse proteins by phosphorylation. GSK3β is known to be involved in multiple pivotal signaling pathways and has been shown to play a role in development, cell polarity, insulin signaling, metabolic regulation, neurodegenerative disorders, and cancer [25], [27], [49]–[51]. Moreover, recent evidence suggests that GSK3β can influence RNA splicing [52]. Considering the shift in isoform expression observed in our experiments, it can be speculated that GSK3β may influence both the transcription and splicing of CAR.

One of the targets of GSK3β is β-catenin, a key mediator of the Wnt signaling pathway and a protein also known to interact and co-localize with CAR at cell junctions [16]. It is through transcriptional regulators, such as β-catenin, Sp1, SNAIL, and SMAD, that GSK3β-mediated regulation has an effect at the transcriptional level. GSK3β is downregulated by CSE although the precise mechanisms are unclear [40]–[41]. We show for the first time that acute SSS exposure downregulates GSK3β. CAR has been shown to be transcriptionally regulated by SP1, SNAIL, and SMAD, thus, we hypothesized that GSK3β would play an important role in the regulation of CAR expression. Treatment of polarized CaLu-3 cells for 18 h with a GSK3β inhibitor resulted in the upregulation of CAR mRNA and protein, and CAR^Ex8^ in particular, similar to that resulting from SSS exposure. GSK3β inhibition also resulted in a similar degree of increased adenovirus transduction as SSS exposure. Future work will focus on elucidating the mechanism behind the down regulation of GSK3β and whether this directly affects the interaction between CAR and β-catenin, activation of SP1, SNAIL, and/or SMAD, as well as a potential influence on splicing. GSK3β inhibitors are under intense clinical investigation as therapeutics for several diverse diseases such as diabetes, depression, and neurodegeneration [28], [35]. Therefore studies such as these may expand the range of applications for clinically relevant therapeutics to novel uses or lead to a greater understanding of potential side effects.

Inflammation is known to play an important role in the development of COPD and both genetic and environmental factors have been implicated [53]–[54]. Moreover, childhood respiratory illness is a risk factor for COPD [55]–[56]. Interestingly, studies link chronic adenovirus infection with the development or progression of COPD [8]–[12]. Taken together with our data, SSS exposure may lead to increased airway susceptibility to adenoviral infection and hence an increase in opportunities to develop chronic adenovirus infection. This in turn may potentially lead to or exacerbate symptoms by increasing the inflammatory response and lead to disease progression. Future studies will investigate the important potential link between ETS exposure, increased susceptibility to adenovirus infection, and COPD progression.

Previous studies using a similar air-liquid interface system have primarily focused on MSS [21], [57]–[58]. For example, kinase inhibition studies in polarized CaLu-3 cells have shown that the acute loss in barrier function due to MSS exposure is likely a regulated process [21], [57]. A similar loss in barrier function is observed in SSS exposure and it is possible that a similar acute effect occurs. However, despite similar tight junction recovery in all conditions, SSS-exposure specifically increases the susceptibility of epithelia to apical adenovirus infection 18 h post-exposure indicating that other longer-lasting changes occur in SSS-exposed epithelia. To our knowledge, only one study has investigated SSS exposure on airway cells. Analysis of SSS exposure on bronchial epithelial cells, HFBE, demonstrated dose-dependent toxicity and an inverse correlation with glutathione content [31]–[32]. No comparison to MSS was made in this study. A few studies have attempted *in vivo* SSS exposure in animal models [59]–[61]. Significant changes, including increased inflammatory cytokine secretion, systemic lipid peroxidation, and myocardial infarct size, but decreased heart contractile function and vitamin E levels [59]–[60]. Moreover, studies of secondhand smoke *in vivo*, have shown increased secretion of proinflammatory cytokines, such as IL-8 [61]. Interestingly, Lutschg *et al.* have recently shown that acute exposure of polarized human bronchial epithelial 16HBE14o cells to recombinant IL-8 leads to increased apical CAR and adenoviral infection [62]. Although the isoform specificity of CAR was not investigated, it could be speculated that this was a shift of CAR^Ex8^ from a subapical region to the apical surface. We found that IL-8 and MCP-1 were upregulated at 18 h post-SSS exposure (data not shown). This would be consistent with GSK3β inhibition resulting in transcriptional upregulation of CAR, coupled with a cellular response to elevated IL-8 secretion causing newly synthesized CAR^Ex8^ to localize at the apical surface, resulting in the increased susceptibility of the airway to adenoviral infection. Additional cellular signaling pathways are likely to be involved andthis should be a subject of future investigations.

In summary, we show for the first time that acute SSS exposure, but not MSS exposure, significantly increases the susceptibility of polarized lung epithelial cells to adenoviral infection. We also show that SSS exposure significantly downregulates the pivotal cellular kinase GSK3β, and that direct inhibition of GSK3β in polarized CaLu-3 cells has a similar effect on CAR expression and adenoviral infection as acute SSS exposure (Figure 9). These studies provide novel insight into the difference in biological responses that occur during CS exposure and evidence that cellular, in addition to immunologic, mechanisms play a significant role in the susceptibility of the airway epithelium to viral infection.

## Supporting Information

Figure S1 **Schematic representation of the smoke collection and exposure method.** The Walton automatic smoke machine was connected directly to the the cultex exposure system via tubing. Not drawn to scale. Filtered control air and mainstream or sidestream smoke paths. Regulated, compressed air feeds the Walton smoker, which operates by solenoid valves switching air flow under the control of a timer. Smoke or plain air (purge) is directed into the reservoir at intervals described in the methods, and routed to the Cultex chamber, which contains two groups of three Transwells. The filtered air control path begins with pump A pushing air through a HEPA filter, bubbler, and Cultex chambers. Output from both sets of Cultex chambers is drawn through flow controllers at 8.3 ml/Transwell/min to the vacuum reservoir evacuated by pump B.
(TIF)

Figure S2 **CAR expression is increased and localization is altered in polarized CaLu-3 cells 18 h post-MSS exposure.** A: Representative schematic showing the expected localization of ezrin, ZO-1, and CAR in polarized cells. Immunofluorescence staining of B: total endogenous CAR (green) and B: CAR^Ex8^ (green), co-stained with antibodies directed against either the tight junction protein ZO-1 (red) or the apical protein ezrin (red), in polarized CaLu-3 cells 18 h after exposure to MSFA or MSS. Nuclei are counterstained with DAPI (blue). Representative X-Z sections are shown from three independent experiments. Dotted white line represents the Transwell filter that cells are seeded on. Black line = 10 µm. Confocal microscopy (60× oil immersion).
(TIF)
