# Supplementary figures and images for: Sidestream Smoke Exposure Increases the Susceptibility of Airway Epithelia to Adenoviral Infection
Source: PLoS One. 2012 Nov 15;7(11):e49930. doi: 10.1371/journal.pone.0049930 (PMC3499494; doi:10.1371/journal.pone.0049930)

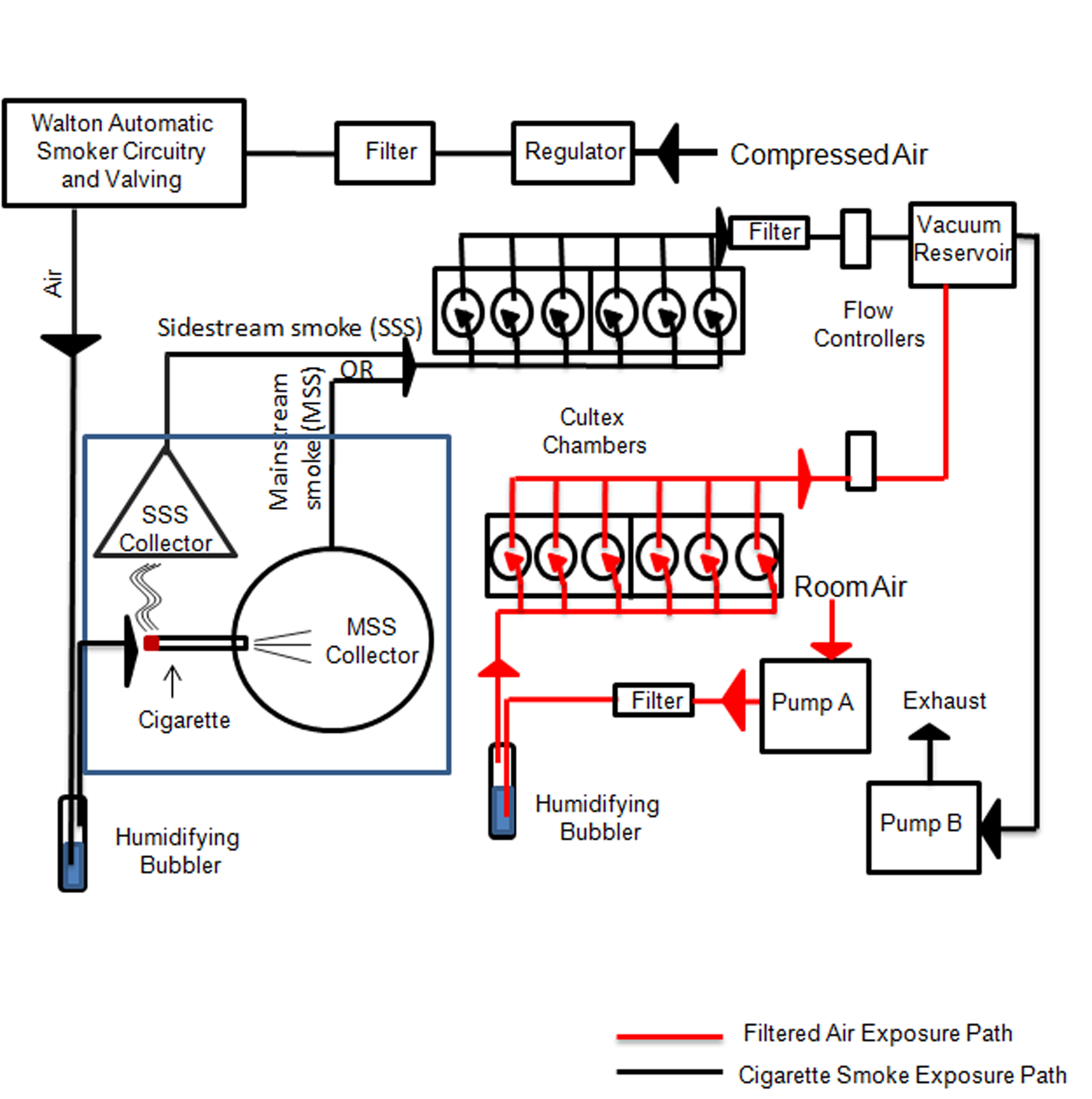

Supplement: Figure S1 — Schematic representation of the smoke collection and exposure method. The Walton automatic smoke machine was connected directly to the the cultex exposure system via tubing. Not drawn to scale. Filtered control air and mainstream or sidestream smoke paths. Regulated, compressed air feeds the Walton smoker, which operates by solenoid valves switching air flow under the control of a timer. Smoke or plain air (purge) is directed into the reservoir at intervals described in the methods, and routed to the Cultex chamber, which contains two groups of three Transwells. The filtered air control path begins with pump A pushing air through a HEPA filter, bubbler, and Cultex chambers. Output from both sets of Cultex chambers is drawn through flow controllers at 8.3 ml/Transwell/min to the vacuum reservoir evacuated by pump B. (TIF) [file pone.0049930.s001.tif]

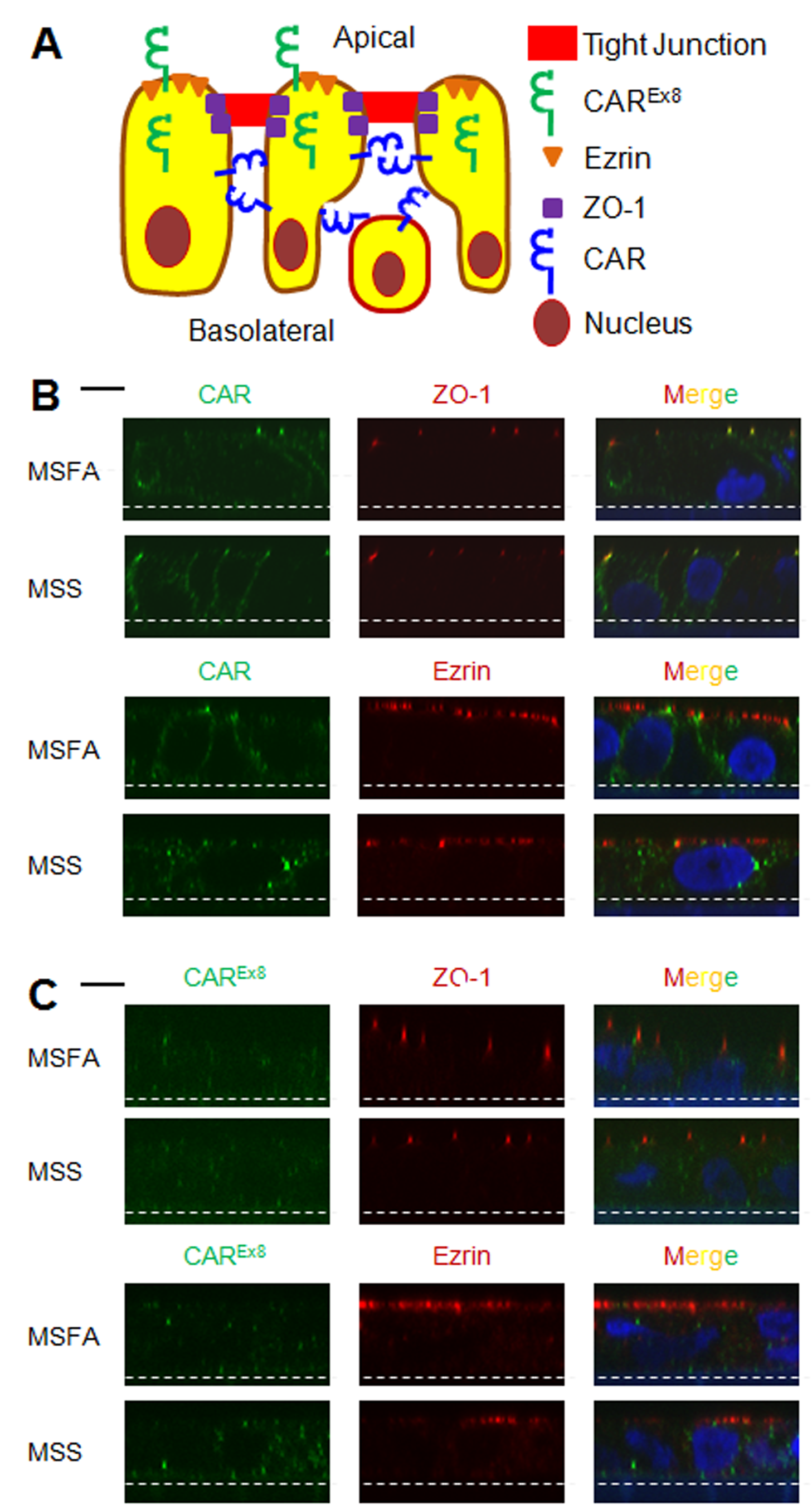

Supplement: Figure S2 — CAR expression is increased and localization is altered in polarized CaLu-3 cells 18 h post-MSS exposure. A: Representative schematic showing the expected localization of ezrin, ZO-1, and CAR in polarized cells. Immunofluorescence staining of B: total endogenous CAR (green) and B: CAREx8 (green), co-stained with antibodies directed against either the tight junction protein ZO-1 (red) or the apical protein ezrin (red), in polarized CaLu-3 cells 18 h after exposure to MSFA or MSS. Nuclei are counterstained with DAPI (blue). Representative X-Z sections are shown from three independent experiments. Dotted white line represents the Transwell filter that cells are seeded on. Black line = 10 µm. Confocal microscopy (60× oil immersion). (TIF) [file pone.0049930.s002.tif]
